# Lipase-Catalyzed Synthesis, Properties Characterization, and Application of Bio-Based Dimer Acid Cyclocarbonate

**DOI:** 10.3390/polym10030262

**Published:** 2018-03-03

**Authors:** Xin He, Guiying Wu, Li Xu, Jinyong Yan, Yunjun Yan

**Affiliations:** Key Laboratory of Molecular Biophysics of the Ministry of Education, College of Life Science and Technology, Huazhong University of Science and Technology, Wuhan 430074, China; n785888@163.com (X.H.); wuguiying@hust.edu.cn (G.W.); xuli@hust.edu.cn (L.X.); yjiny@126.com (J.Y.)

**Keywords:** glycerol carbonate, dimer acid, esterification, lipase, cyclocarbonate, bio-based non-isocyanate polyurethane

## Abstract

Dimer acid cyclocarbonate (DACC) is synthesized from glycerol carbonate (GC) and *Sapium sebiferum* oil-derived dimer acid (DA, 9-[(*Z*)-non-3-enyl]-10-octylnonadecanedioic acid). Meanwhile, DACC can be used for synthetic materials of bio-based non-isocyanate polyurethane (bio-NIPU). In this study, DACC was synthesized by the esterification of dimer acid and glycerol carbonate using Novozym 435 (*Candida antarctica* lipase B) as the biocatalyst. Via the optimizing reaction conditions, the highest yield of 76.00% and the lowest acid value of 43.82 mg KOH/g were obtained. The product was confirmed and characterized by Fourier transform-infrared spectroscopy (FTIR) and nuclear magnetic resonance spectroscopy (NMR). Then, the synthetic DACC was further used to synthesize bio-NIPU, which was examined by FTIR, thermogravimetric analysis (TGA), and differential scanning calorimetry (DSC), indicating that it possesses very good physio-chemical properties and unique material quality with a potential prospect in applications.

## 1. Introduction

**Figure 1:**
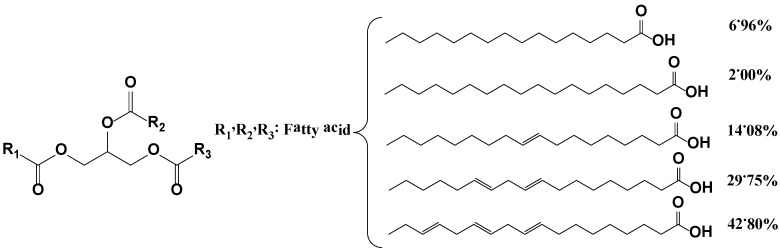
The structure of *Sapium sebiferum* oil and its fatty acid content.

Forestry oil has become an important renewable resource in the consideration of environmental concerns, fossil fuel depletion, and food production [1,2]. The major component of forestry oil is triglycerides (esters of glycerol with three fatty acids); there are different long-chain fatty acids in the different sources of oil [3], which can provide different applications in industry. Since *Sapium sebiferum* oil, a non-edible oil, is abundant in China and its production significantly benefits the environment construction, it has been attracting wide attention and has been moderately studied [4,5]. Moreover, *S. sebiferum* can give way to arable land because it can grow in alkaline, saline, droughty, and acidic soil. Its seeds contain 45–60% oil, which is mostly unsaturated, resulting in a high iodine value of 186.8 g of I_2_/100 g. *S. sebiferum* oil possesses many double bonds (Figure 1), which are the appropriate groups for the synthesis of various industrial compounds and polymers, especially for the synthesis of dimer acids [5,6].

Dimer acids (DAs), produced by a Diels–Alder reaction of unsaturated fatty acids from unsaturated oil, are highly value-added industrial products and are widely used in different fields, such as adhesives, preservatives, plastic additives, and lubricants [7,8,9]. On the other hand, glycerol can also be easily produced from forestry oil, or sometimes is a byproduct of forestry oil processing [10,11,12]. A downstream synthetic product of glycerol is glycerol carbonate (GC), which also has many different industrial applications, such as coatings, surfactants, cosmetics, lubricants, and so on. Its structure contains a 2-oxo-1,3-dioxolane group and a hydroxyl group, which have high reaction activity with anhydrides, acyl chlorides, isocyanates, and the like [13,14,15]. Having a bio-based origin and wide reactivity, GC has become a versatile and renewable building block for chemical synthesis. To obtain highly purified GC, different synthesis methods have been investigated, including direct synthetic routes and indirect synthetic routes [16,17]. They all aim at the lowest production cost, the least pollution, and the highest yield of GC.

At present, GC has been employed as a substrate to synthesize non-isocyanate polyurethane (bio-NIPU) in order to meet the increasingly serious environment regulations. The route is green and without the requirement of either toxic isocyanate monomers or phosgene [18]. However, the main synthesis pathway of cyclocarbonate is based on the catalytic synthesis of *N*,*N*’-dicyclohexylcarbodiimide (DCC) and 4-dimethylaminopyridine (DMAP), which are also poisonous to humans in some way [18,19,20]. Consequently, the cyclocarbonates react with amines to form urethane, or more specifically hydroxyurethane bonds [21,22]. The consumption and the recycling of the catalysts in the synthesis reaction is a huge problem, especially in large-scale industrial applications.

**Scheme 1:**
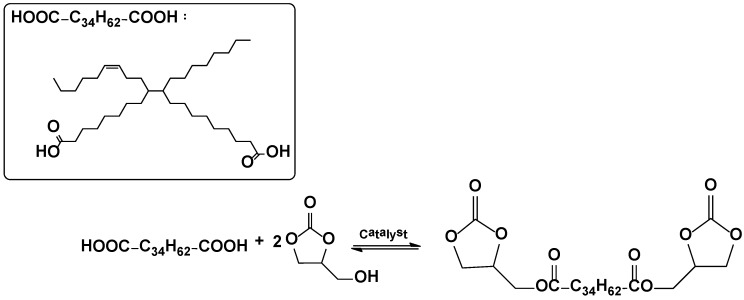
Synthesis of dimer acid cyclocarbonate (DACC).

Dimer acid cyclocarbonate (DACC) is a monomer of non-isocyanate polyurethane. It has the following advantages: (1) simple synthetic methods; (2) biodegradability; and (3) biologic material. The route of synthesis is shown in Scheme 1. The traditional chemical synthesis of DACC requires a large number of chemical catalysts and dehydrants, resulting, in most cases, in numerous byproducts. Until now, most cyclocarbonates are chemically synthesized, which needs high pressure and more energy consumption and produces toxic waste [23,24]. On the contrary, enzymatic synthesis has garnered considerable interest with regard to its many merits, such as high regio-, chemo- and enantio-selectivity, environmental friendliness, mild reaction conditions, simple purification steps, and the enzyme being reused [16,25,26]. So far, there are considerable researches on the enzymatic synthesis of ester compounds [27]. Carlos et al. obtained the dodecyl lactate and glycolate through a lipase from *Candida antarctica* [28]. Afife et al. investigated isoamyl acetate synthesis using immobilized *Rhizomucor miehei* and *C. antarctica* lipases by esterification of acetic acid and isoamyl alcohol without organic solvent [29]. Actually, enzymatic synthesis of ester compounds is becoming a research hotspot. Novozym 435 (*Candida antarctica* lipase B physically immobilized within a macroporous resin of poly(methyl methacrylate)), a commercially available heterogeneous biocatalyst, has been used successfully for polyesters and polyamides synthesis [30].

Therefore, in this work, the enzymatic synthesis of DACC was first investigated by esterification of bio-based DA with GC. Then, experiments were designed to optimize the conditions of DACC synthesis, mainly to examine the effects of GC/DA molar ratio, reaction time, enzyme concentration, reaction temperature, molecular sieve content, agitation speed, solvent and enzyme cycling. The derivative DACC was subsequently reacted with different amines to prepare various bio-based non-isocyanate polyurethanes (bio-NIPUs), and their chemical structures and thermal properties were further characterized.

## 2. Materials and Methods

### 2.1. Materials

Dimer acid (DA, 9-[(*Z*)-non-3-enyl]-10-octylnonadecanedioic acid, CAS No. 61788-89-4) and glycerol carbonate (GC, CAS No. 931-40-8) were purchased from Bangcheng Chemical Ltd. (Shanghai, China) and Tokyo Chemical Industry Co., Ltd. (Tokyo, Japan), respectively. Novozym 435 (immobilized lipase B from *C. antarctica*) with a specific activity 10,000 propyl laurate units (PLUs) per gram was commercially obtained from Novozym Co. Ltd. (Zealand, Denmark). Acetonitrile (CAS No. 75-05-8), hexane (CAS No. 110-54-3), dichloromethane (CAS No. 75-09-2), molecular sieve type 4A (CAS No. 70955-01-0), ethylenediamine (EDA, CAS No. 107-15-3), diethylenetriamine (DETA, CAS No. 111-40-0), triethylenetetramine (TETA, CAS No. 112-24-3), tetraethylenepentamine (TEPA, CAS No. 112-57-2), and hexanediamine (HMDA, CAS No. 124-09-4) were analytical reagents and bought from Sinopharm Chemical Reagent Ltd. Co. (Shanghai, China).

### 2.2. Synthesis of Dimer Acid Cyclocarbonate (DACC)

DACC was synthesized in acetonitrile via an esterification reaction between DA and GC catalyzed by Novozym 435 [31,32,33,34,35]. Experiments were conducted in a conical flask placed in a thermostat shaking bed with a temperature monitor. A single factorial experiment was first designed. The effects of GC/DA molar ratio (2.00:1.00–10.00:1.00), time (4–24 h), Novozym 435 concentration (1–10 wt %, *w*/*w* DA), temperature (35–65 °C), molecular sieve content (30–100 wt %, *w*/*w* DA), agitation speed (50–300 rpm), solvent (acetonitrile, tert-butanol, tetrahydrofuran, acetone, and methylbenzene), and Novozym 435 cycle number (1–5 times) on the production of DACC were explored. The yield and acid value were chosen as the indicators.

### 2.3. Purification of the Esters

All the esters were purified after being prepared. These purified materials were further used in follow-up experiments to characterize their structures and calculate the conversion rates [28].

The enzyme was removed from the reaction mixture via filtration and the solvent was evaporated using a rotovap system. Then, the filtered and evaporated reaction mixtures were dissolved in *n*-hexane and the esters precipitated out, aiming to separate DA. Finally, precipitation in deionized water afforded DACC as a yellow viscous liquid; the residual water from the production was evaporated.

### 2.4. Synthesis of Bio-NIPU

DACC and dichloromethane were placed in the Teflon mold and stirred mechanically for 5 min. Then, different amines (EDA, DETA, TETA, TEPA, and HMDA) were added and the mixture was stirred mechanically for 5 min [36]. Thereafter, the mold was heated at 90 °C and reduced pressure for 12 h. All products were kept in a desiccator for later use.

### 2.5. Analysis and Characterization

The FTIR spectra of the samples were recorded in the frequency range of 4000–400 cm^−1^ with a spectral resolution of 4 cm^−1^ using a Bruker Vertex70 FTIR spectrometer equipped with a DTGS detector (Bruker Optics, Karlsruhe, Germany). Taking the KBr plate as a blank for the background, a few of the samples were dropped on it for the test.

The ^1^H-NMR spectra of the samples were determined by a Bruker AV600 MHz NMR spectrometer (Bruker, Karlsruhe, Germany). The samples were dissolved in CDCl_3_ (Tetramethylsilane (TMS) as internal standard) and then placed in 5 mm diameter NMR sample tubes for the determination. The measurement was performed at room temperature.

To confirm the degree of esterification, samples were characterized by acid value according to Chinese National Standard (GB/T 264-1983) [37]. The yield was calculated by the following equation. The mass of theoretical objective product was calculated according to the starting mass of DA. The actual objective product was weighed accurately after purification.

$$\mathrm{Product}\;\mathrm{yield}=\frac{\mathrm{the}\;\mathrm{weight}\;\mathrm{of}\;\mathrm{actual}\;\mathrm{objective}\;\mathrm{product}}{\mathrm{the}\;\mathrm{weight}\;\mathrm{of}\;\mathrm{theoretical}\;\mathrm{objective}\;\mathrm{product}}\times 100\%$$

Thermogravimetric analyses (TGA) were performed using a Pyris 1 TGA (Perkin-Elmer Instruments, Boston, MA, USA) at a heating rate of 10 °C /min. Approximately 5 mg of sample was subjected to temperatures from 50 to 600 °C in an N_2_ atmosphere.

Differential scanning calorimetry (DSC) analysis was performed on a Perkin-Elmer Diamond DSC instrument (Perkin-Elmer Instruments, Boston, MA, USA) under N_2_ atmosphere. The sample was first heated from −50 to 100 °C at 10 °C/min.

## 3. Results and Discussion

### 3.1. Single Factorial Experiments

#### 3.1.1. Effect of GC/DA Molar Ratio

**Figure 2:**
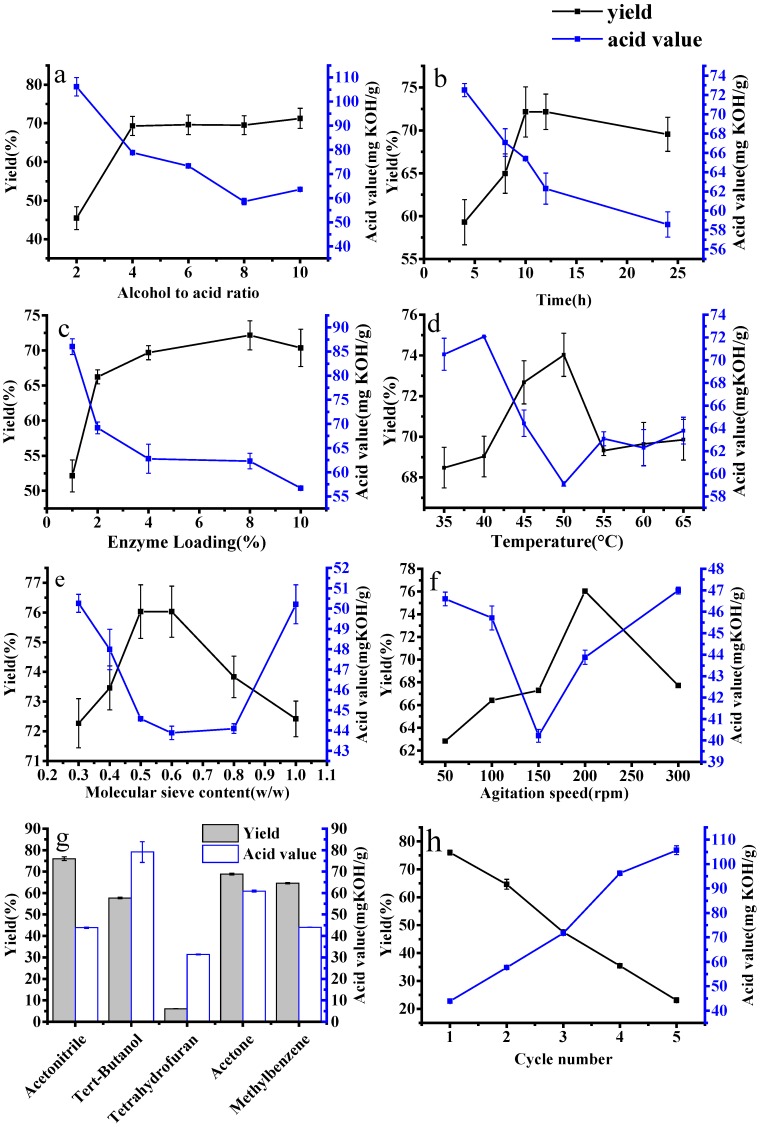
Effect of single factorial experiments. (**a**) GC/DA molar ratio, (**b**) time, (**c**) enzyme concentration, (**d**) temperature, (**e**) molecular sieve content, (**f**) agitation speed, (**g**) solvent, (**h**) cycle number.

According to the chemical equation in Scheme 1, 1 mol of DACC is synthesized from 1 mol of DA and 2 mol of GC through esterification, and 2 mol water is obtained. However, according to this theoretical ratio, raw materials cannot completely react as a result of the reversible reaction. Meanwhile, DA has a long alkyl chain, resulting in steric hindrance. This repulsive hindrance lowers the electron density in the intermolecular region and disturbs the bonding interactions [38]. Therefore, with an increase in the ratio, the reaction is driven in the direction of a forward reaction. Therefore, the effect of the GC/DA molar ratio was optimized for DACC synthesis (Figure 2a). As shown in Figure 2a, the DACC yield increased with the molar ratio from 2.00 to 10.00, which was attributed to more collisions between DA and GC. When the molar ratio reached 4.00, the DACC yield remained about the same with increasing molar ratio. However, the acid value decreased, which was ascribed to the decrease in carboxylic acid (–COOH) groups. When the molar ratio reached 10.00, the acid value showed little change. Considering the economy of the raw materials and that a lower acid value led to a higher esterification degree, a GC/DA molar ratio of 8.00 was a better choice. Although the theoretical GC/DA molar ratio for synthesis of DACC was 2.00, its actual optimized GC/DA molar ratio for DACC synthesis, which resulted in a better yield and a lower acid value, was 8.00. The following tests used this optimal GC/DA molar ratio.

#### 3.1.2. Effect of Time

To find the proper reaction time, the reaction was performed from 4 to 24 h. As shown in Figure 2b, the reaction time had a great effect on yield. As the reaction time went on, the yield continued to increase. However, the reaction showed little change after 10 h. When the reaction time reached 24 h, the yield did not change too much because an equilibrium had been reached. Therefore, 12 h with the highest yield was selected as the optimal time. Although it had the same yield as at 10 h, the acid value was higher than that of 12 h. Given the cost of time, 12 h was better than 24 h even though the acid value was lower. Therefore, 12 h was determined as the optimal reaction time for maximizing the efficiency.

#### 3.1.3. Effect of Enzyme Concentration

Enzymes are more efficient and environmentally friendly catalysts than chemical catalysts. Here, Novozym 435 was employed to catalyze DACC synthesis. The effect of enzyme concentration and its optimal dosage were investigated at various levels ranging from 1 to 10 wt % (Figure 2c). It can be seen from Figure 2c that the yield increased as the Novozym 435 concentration increased from 1 to 8 wt %. When the Novozym 435 concentration increased to 10 wt %, the yield decreased slightly. The adsorption of DACC on the immobilized enzyme always occurred; after reaching 10 wt %, a higher enzyme concentration led to higher adsorption. However, an excess in enzyme concentration could hamper the internal diffusion and relevant acquaintance of the substrate with the active sites [39]. Therefore, the increased concentration of immobilized enzyme was good for the synthesis of DACC, leading to a lower acid value, while the yield was affected with high enzyme dosage. Considering the better yield and an appropriate acid value, a Novozym 435 concentration of 8 wt % was chosen as the optimal enzyme concentration.

#### 3.1.4. Effect of Temperature

The reaction temperature exerts a significant effect on enzyme activity and the economic effect of the DACC synthesis [40]. The effect of temperature on the esterification was investigated in the range from 35 to 65 °C (see Figure 2d). The maximum yield and minimum acid value were both obtained at 50 °C. A higher reaction temperature could increase the reaction rate and yield but it would lead to enzyme inactivation. At a lower reaction temperature, the substrate and intermediate cannot dissolve in sufficient quantity for synthesis [41,42]. Hence, the reaction temperature of 50 °C was selected as the optimal temperature.

#### 3.1.5. Effect of Molecular Sieve Content

Because hydrolysis is the reverse reaction of esterification, removing water is the key to the degree of esterification. The effect of molecular sieve content was investigated over a range from 30 to 100 wt % (Figure 2e). The results show that yield reached a maximum value at 50 and 60 wt %, while the acid value reached a minimum at 60 wt %. The presence of the molecular sieves increased the yield at the initial stage because the desiccant can urge the reaction to esterification instead of hydrolysis. However, the yield decreased from 60 to 100 wt % and this could have been caused by molecular sieve adsorption. GC was adsorbed and diminished the effective concentration in the reaction. Meanwhile, the acid value increased from 60 to 100 wt %, indicating that molecular sieves adsorbed GC rather than DA. Considering the water absorption and raw materials adsorption of molecular sieves, the content at 60 wt % was defined as the optimal condition.

#### 3.1.6. Effect of Agitation Speed

In this reaction, agitation speed is an easily overlooked single factor. Figure 2f shows that the yield reached a maximum when the agitation speed attained 200 rpm. From 50 to 200 rpm, the yield continued to increase because the mechanical agitation accelerated the collisions of the molecules. However, the yield decreased when the agitation speed approached 300 rpm; a possible reason could be that a high agitation speed would reduce the contact time and the contact area with the reaction interface. There was an interesting phenomenon that the acid value reached a minimum value when the agitation speed was 150 rpm. A reasonable explanation is that high agitation speeds could hamper the reaction between DA and the second GC, which then led to high acid values between agitation speeds of 150 and 300 rpm. Considering the yield and the acid value, 200 rpm was set as the appropriate agitation speed.

#### 3.1.7. Effect of Solvent

In this reaction, dimer acid and glycerol carbonate do not mix. Therefore, a suitable solvent should dissolve enough raw materials for the lipase-catalyzed esterification, and the solvent should not affect lipase activity and stability. The results show that the reaction had the lowest yield in tetrahydrofuran, which might have altered the native conformation of the lipase (Figure 2g). Tert-butanal and acetone led to high acid values because GC did not dissolve entirely, which would have led to inadequate participation in the reaction. However, acetonitrile did dissolve the DA and GC at 50 °C, and had a low effect on Novozym 435. Foremost, the yield had the highest value and the acid value was low enough in acetonitrile. Thus, acetonitrile was selected for use in subsequent experiments.

#### 3.1.8. Reuse of Novozym 435

Immobilized lipase has a reusable feature. We performed a series of experiments to examine its characteristic for the synthesis of DACC. After one cycle, the immobilized lipase was recovered by paper filtration and then dried for the next cycle. As shown in Figure 2h, the yield decreased and the acid value increased with the cycle number. The results indicate that the immobilized lipase loses its activity with cycle number; the probable reason could be that dichloromethane, which was used to wash the immobilized lipase in order to clear the remaining raw materials and DACC, could cause enzyme inactivation. Therefore, although the first two cycles can provide sufficient objective product, improving cycle efficiency will be a key research direction in the future.

### 3.2. Determination of DACC by FTIR

**Figure 3:**
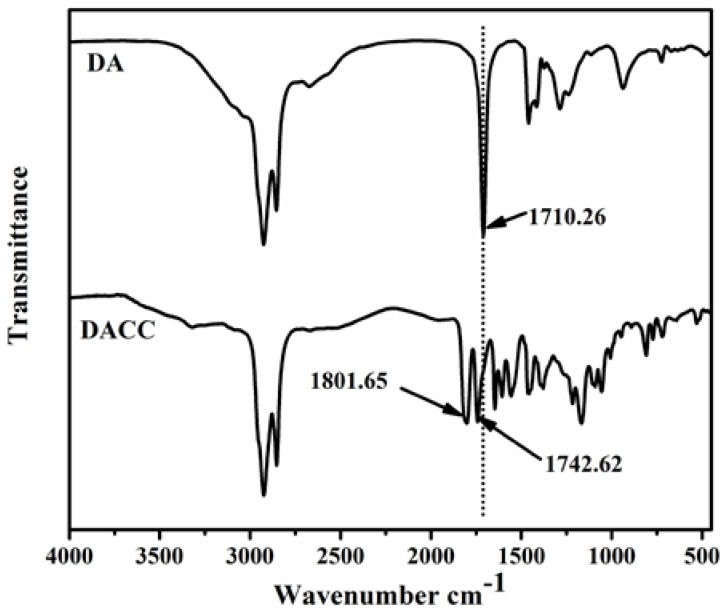
FTIR spectra of DA and DACC.

Figure 3 shows the FTIR spectra of DA and DACC. The top curve is the FTIR spectra of DA, which had a carbonyl C=O stretching vibration at 1710.26 cm^−1^ attributed to a carboxyl group. The curve below is the FTIR spectra of DACC, which had two carbonyl C=O stretching vibrations attributed to an ester group at 1742.62 cm^−1^ and carbonic ester at 1810.65 cm^−1^. Obviously, the carboxyl group disappeared and two new carbonyl C=O stretching vibrations appeared because the carboxyl group reacted with a hydroxyl group to get an ester group. The FTIR spectra of DA and DACC clearly indicate the formation of DACC, especially the changes of the different carbonyl C=O stretching vibrations.

### 3.3. Determination of DACC by ^1^H-NMR

**Figure 4:**
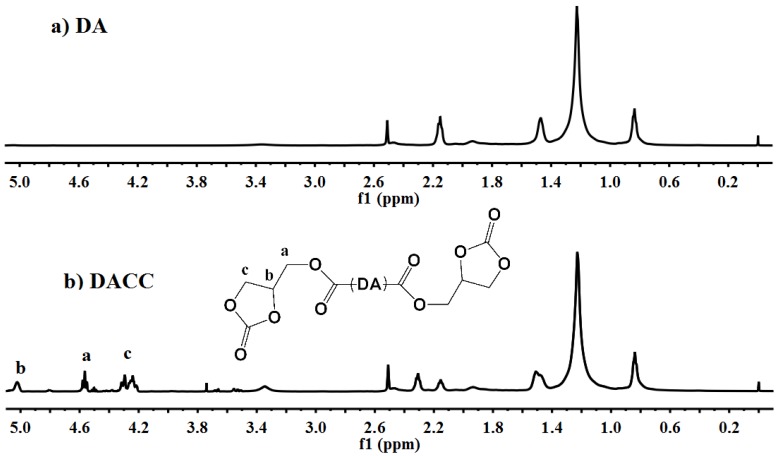
The ^1^H-NMR spectra of DA (**a**) and DACC (**b**).

Compared with the spectra of DA (Figure 4a), the spectra of DACC (Figure 4b) included some new peak groups attributed to methylene protons and methine protons [43], which were δ 4.21–4.28 (c, –OCH_2_CH–, 2H), δ 4.29–4.34, 4.56–4.59 (a, –CHCH_2_O–, 2H), δ 4.99–5.03 (b, –CH–, 1H), suggesting that the DACC had been successfully synthesized.

### 3.4. Characterization of Bio-NIPU

#### 3.4.1. Characterizing Bio-NIPU by FTIR

**Scheme 2:**
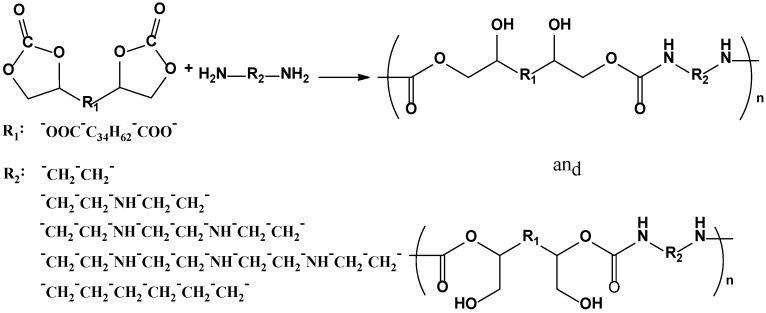
Synthesis of bio-NIPU.

**Figure 5:**
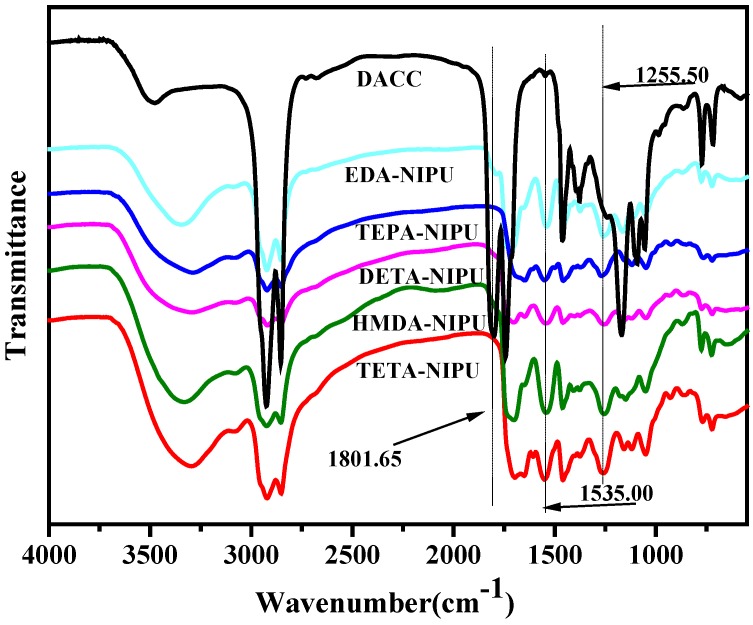
FTIR spectra of different NIPUs and DACC.

Figure 5 shows the FTIR spectra of different bio-NIPUs and DACC. Compared with the FTIR spectra of DACC, the bio-NIPU curves had some differences. The peak at 1801.65 cm^−1^ only existed in DACC because it is the carbonyl C=O stretching vibration from carbonic ester. As can be seen from Scheme 2, the cyclocarbonates were opened by the amine groups. Two new peaks appeared at 1535.00 and 1255.50 cm^−1^, which are the N–H stretching vibration and C–N stretching vibration, respectively, in bio-NIPU. The results confirm successful synthesis of bio-NIPU by DACC and different amines.

#### 3.4.2. Determination of Bio-NIPU via DSC

**Figure 6:**
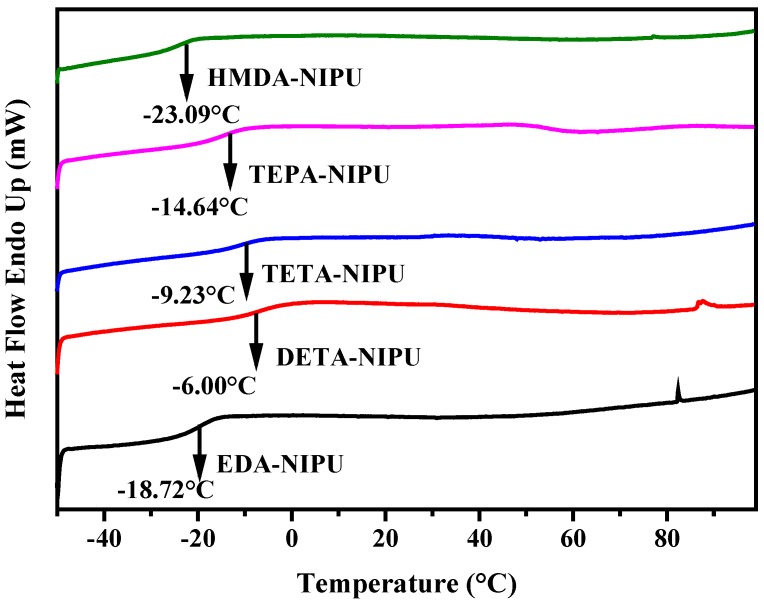
DSC curves of different NIPUs.

To study the thermal properties of different bio-NIPUs, the DSC curves are recorded in Figure 6. Glass transition temperature (*T*_g_) values of the bio-NIPUs with secondary amines were higher than those of the bio-NIPUs with diamine. *T*_g_ value of DETA-NIPU was −6.00 °C, which was higher than the other NIPUs. The results suggest that secondary amines have a positive effect on the crystallization of bio-NIPU. These bio-NIPUs have low *T*_g_ values due to the soft segment long chain structure of dimer acid. There was only one single glass transition temperature for all bio-NIPUs, indicating good miscibility. 

#### 3.4.3. Determination of Bio-NIPU via TGA

**Figure 7:**
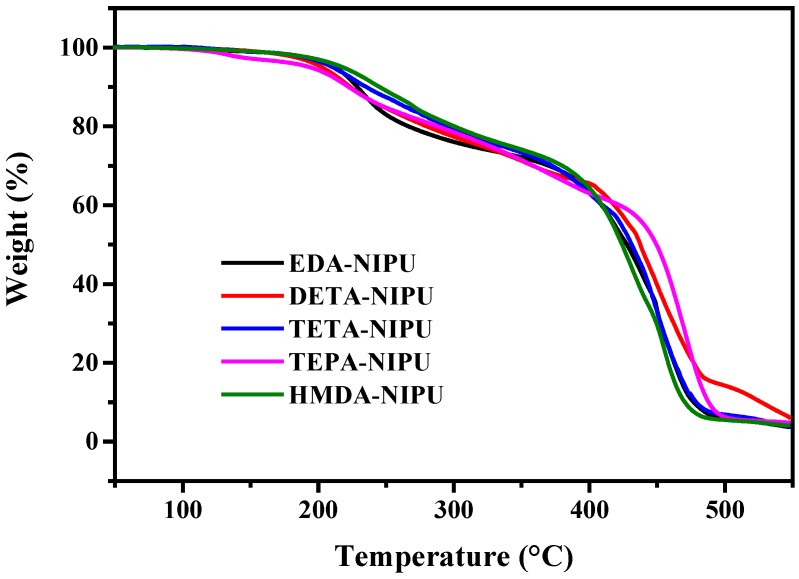
TGA curves of different NIPUs.

**Figure 8:**
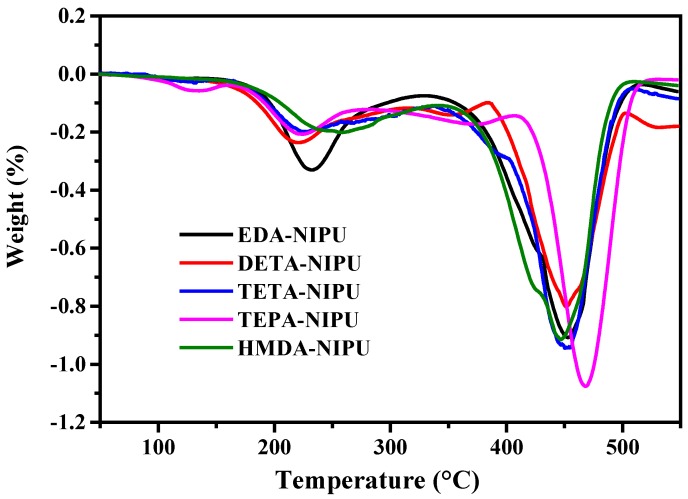
DTG curves of different NIPUs.

The influence of the average amine functionality was investigated for the thermal stability of the synthesized polymers via thermogravimetric analysis (TGA). Figure 7 and Figure 8 present TGA and differential thermal gravity (DTG) curves for different bio-NIPUs, respectively. The thermal stability of the EDA-NIPU, DETA-NIPU, TETA-NIPU, and HMDA-NIPU were similar with *Td*_5%_ (the temperature at 5% weight loss) around 210 °C. The thermal stability value of TEPA-NIPU was lower with a *Td*_5%_ equal to 194 °C (Table 1). The char contents at 550 °C are also shown in Table 1. These results suggest that secondary amines have no contribution to bio-NIPU synthesis. Thermal degradation of different bio-NIPUs takes place in two stages, corresponding to the hard and soft segments, because of thermodynamic incompatibility of the two segments in the bio-NIPU matrix. The temperatures for the maximum rate of degradation (*T*_max_) for each of the two stages for different bio-NIPUs are given in Figure 8 and Table 1. The *T*_max1_ values of DETA-NIPU, TETA-NIPU, and TEPA-NIPU were about 220 °C. However, *T*_max1_ values of EDA-NIPU and HMDA-NIPU were higher due to no reaction between the secondary amines and cyclocarbonates. The *T*_max2_ value of TEPA-NIPU was 468 °C, which was the highest temperature in the different bio-NIPUs. This result is due to the high boiling point of TEPA, which may influence the maximum rate of degradation of the soft segments. 

## 5. Conclusions

This study attempted to optimize the enzymatic catalyzed production of DACC from DA and GC derived from *S. sebiferum* oil by esterification using Novozym 435 as the catalyst. Considering the economic effect and efficiency criteria, GC/DA molar ratio, reaction time, Novozym 435 concentration, reaction temperature, molecular sieve content, agitation speed, and different solvents were optimized one by one. Meanwhile, enzyme cycling was studied. The yield was 76.00% and the lowest acid value for synthesized DACC was 43.82 mg KOH/g under optimal conditions. The product was further confirmed by FITR and NMR. Then, DACC was employed to synthesize bio-NIPU and subsequently characterized by FTIR, DSC, and TGA. These studies prove that it is feasible to synthesize DACC by lipase. Furthermore, the bio-NIPU had a low *Tg* and a good thermostability, which can be potentially used in the coating industry. This study demonstrates a new method to synthesize cyclocarbonate, which has a prosperous future potential for the bio-polyurethane industry.

## Figures and Tables

**Table 1:** TGA and DTG results for different bio-NIPUs.

	EDA-NIPU	DETA-NIPU	TETA-NIPU	TEPA-NIPU	HMDA-NIPU
*Td*_5%_ (°C)	212.31	202.90	210.85	194.09	218.10
*T*_max1_ (°C)	232.68	220.58	227.81	224.02	258.71
*T*_max2_ (°C)	453.16	451.39	450.18	468.00	447.05
Residue at 550 °C (%)	3.7	6.2	3.9	4.9	4.1

## References

[1] Meher L., Vidyasagar D., Naik S. (2006). Technical aspects of biodiesel production by transesterification—A review. Renew. Sust. Energ. Rev..

[2] Monteil-Rivera F., Phuong M., Ye M., Halasz A., Hawari J. (2013). Isolation and characterization of herbaceous lignins for applications in biomaterials. Ind. Crop. Prod..

[3] Miao S., Wang P., Su Z., Zhang S. (2014). Vegetable-oil-based polymers as future polymeric biomaterials. Acta Biomater..

[4] Wu G., Fan Y., He X., Yan Y. (2015). Bio-polyurethanes from Sapium sebiferum oil reinforced with carbon nanotubes: Synthesis, characterization and properties. RSC Adv..

[5] Wu G., He X., Yan Y. (2017). Lipase-catalyzed modification of natural Sapium sebiferum oil-based polyol for synthesis of polyurethane with improved properties. RSC Adv..

[6] Liu Y., Xin H., Yan Y., Xu L. (2010). Preparation of dimeric fatty acid methyl esters and their polyamides from biodiesel. J. Beijing Univ. Chem. Technol..

[7] Huang Y., Ye G., Yang J. (2015). Synthesis and properties of UV-curable acrylate functionalized tung oil based resins via Diels-Alder reaction. Prog. Org. Coat..

[8] Kraack H., Deutsch M., Ocko M.B., Pershan P.S. (2003). The structure of organic langmuir films on liquid metal surfaces. Nucl. Instrum. Methods Phys. Res. Sect. B.

[9] Rix E., Grau E., Chollet G., Cramail H. (2016). Synthesis of fatty acid-based non-isocyanate polyurethanes, NIPUs, in bulk and mini-emulsion. Eur. Polym. J..

[10] Nguyen R., Galy N., Singh A.K., Paulus F., Stoebener D., Schlesener C., Sharma S.K., Haag R., Len C. (2017). A Simple and Efficient Process for Large Scale Glycerol Oligomerization by Microwave Irradiation. Catalysts.

[11] Galy N., Nguyen R., Blach P., Sambou S., Luart D., Len C. (2017). Glycerol oligomerization in continuous flow reactor. J. Ind. Eng. Chem..

[12] Galy N., Nguyen R., Yalgin H., Thiebault N., Luart D., Len C. (2017). Glycerol in subcritical and supercritical solvents. J. Chem. Technol. Biot..

[13] Sonnati M.O., Amigoni S., Taffin De Givenchy E.P., Darmanin T., Choulet O., Guittard F. (2013). Glycerol carbonate as a versatile building block for tomorrow: Synthesis, reactivity, properties and applications. Green Chem..

[14] Clements J.H. (2003). Reactive Applications of Cyclic Alkylene Carbonates. Ind. Eng. Chem. Res..

[15] Kühnel I., Saake B., Lehnen R. (2017). Oxyalkylation of lignin with propylene carbonate: Influence of reaction parameters on the ensuing bio-based polyols. Ind. Crops Prod..

[16] Teng W.K., Ngoh G.C., Yusoff R., Aroua M.K. (2014). A review on the performance of glycerol carbonate production via catalytic transesterification: Effects of influencing parameters. Energy Convers. Manag..

[17] Lee K.H., Park C., Lee E.Y. (2010). Biosynthesis of glycerol carbonate from glycerol by lipase in dimethyl carbonate as the solvent. Bioprocess Biosyst. Eng..

[18] Helou M., Carpentier J.F., Guillaume S.M. (2011). Poly(carbonate-urethane): An isocyanate-free procedure from a,x-di(cyclic carbonate) telechelic poly(trimethylene carbonate)s. Green Chem..

[19] Lu Y., Shen L., Gong F., Cui J., Rao J., Chen J., Yang W. (2012). Polycarbonate urethane films modified by heparin to enhance hemocompatibility and endothelialization. Polym. Int..

[20] Wang X., Li M., Wang T., Jin Q., Wang X. (2014). An improved method for the synthesis of 2-arachidonoylglycerol. Process Biochem..

[21] Carré C., Bonnet L., Avérous L. (2014). Original biobased nonisocyanate polyurethanes: Solvent- and catalyst-free synthesis, thermal properties and rheological behaviour. RSC Adv..

[22] Besse V., Camara F., Méchin F., Fleury E., Caillol S., Pascault J., Boutevin B. (2015). How to explain low molar masses in PolyHydroxyUrethanes (PHUs). Eur. Polym. J..

[23] Bähr M., Bitto A., Mülhaupt R. (2012). Cyclic limonene dicarbonate as a new monomer for non-isocyanate oligo- and polyurethanes (NIPU) based upon terpenes. Green Chem..

[24] Carré C., Bonnet L., Avérous L. (2015). Solvent- and catalyst-free synthesis of fully biobased nonisocyanate polyurethanes with different macromolecular architectures. RSC Adv..

[25] Neta N.S., Teixeira J.A., Rodrigues L.R. (2015). Sugar Ester Surfactants: Enzymatic Synthesis and Applications in Food Industry. Crit. Rev. Food Sci..

[26] Teixeira D.A., Da Motta C.R., Ribeiro C.M.S., de Castro A.M. (2017). A rapid enzyme-catalyzed pretreatment of the acidic oil of macauba (*Acrocomia aculeata*) for chemoenzymatic biodiesel production. Process Biochem..

[27] Singh A.K., Remi N., Galy N., Haag R., Sharma S.K., Len C. (2016). Chemo-Enzymatic Synthesis of Oligoglycerol Derivatives. Molecules.

[28] Torres C., Otero C. (1999). Part I. Enzymatic synthesis of lactate and glycolate esters of fatty alcohols. Enzyme Microb. Technol..

[29] Güven A., Kapucu N., Mehmeto Lu Ü. (2002). The production of isoamyl acetate using immobilized lipases in a solvent-free system. Process Biochem..

[30] Khan A., Sharma S.K., Kumar A., Watterson A.C., Kumar J., Parmar V.S. (2014). Novozym 435-Catalyzed Syntheses of Polyesters and Polyamides of Medicinal and Industrial Relevance. ChemSusChem.

[31] Yao X., Wu G., Xu L., Zhang H., Yan Y. (2014). Enzyme-catalyzed preparation of dimeric acid polyester polyol from biodiesel and its further use in the synthesis of polyurethane. RSC Adv..

[32] Jung H., Lee Y., Kim D., Han S.O., Kim S.W., Lee J., Kim Y.H., Park C. (2012). Enzymatic production of glycerol carbonate from by-product after biodiesel manufacturing process. Enzyme Microb. Technol..

[33] Su E., Zhang M., Zhang J., Gao J., Wei D. (2007). Lipase-catalyzed irreversible transesterification of vegetable oils for fatty acid methyl esters production with dimethyl carbonate as the acyl acceptor. Biochem. Eng. J..

[34] Kim S.C., Kim Y.H., Lee H., Yoon D.Y., Song B.K. (2007). Lipase-catalyzed synthesis of glycerol carbonate from renewable glycerol and dimethyl carbonate through transesterification. Catal. B Enzym..

[35] Ünal M.U. (1998). A Study on the Lipase-Catalayzed Esterification in Organic Solvent. Turk. J. Agric. For..

[36] Cornille A., Auvergne R., Figovsky O., Boutevin B., Caillol S. (2017). A perspective approach to sustainable routes for non-isocyanate polyurethanes. Eur. Polym. J..

[37] Chen L., Liu T., Zhang W., Chen X., Wang J. (2012). Biodiesel production from algae oil high in free fatty acids by two-step catalytic conversion. Bioresour. Technol..

[38] Liu Y., Lotero E., Goodwinjr J. (2006). Effect of carbon chain length on esterification of carboxylic acids with methanol using acid catalysis. J. Catal..

[39] Bansode S.R., Rathod V.K. (2014). Ultrasound assisted lipase catalysed synthesis of isoamyl butyrate. Process Biochem..

[40] Li J., Wang T. (2011). Chemical equilibrium of glycerol carbonate synthesis from glycerol. J. Chem. Thermodyn..

[41] Seong P., Jeon B.W., Lee M., Cho D.H., Kim D., Jung K.S., Kim S.W., Han S.O., Kim Y.H., Park C. (2011). Enzymatic coproduction of biodiesel and glycerol carbonate from soybean oil and dimethyl carbonate. Enzyme Microb. Technol..

[42] Nie K., Xie F., Wang F., Tan T. (2006). Lipase catalyzed methanolysis to produce biodiesel: Optimization of the biodiesel production. J. Mol. Catal. B Enzym..

[43] Huang Y., Pang L., Wang H., Zhong R., Zeng Z., Yang J. (2013). Synthesis and properties of UV-curable tung oil based resins via modification of Diels-Alder reaction, nonisocyanate polyurethane and acrylates. Prog. Org. Coat..

